# Rapid and Highly Sensitive Detection of *Mycobacterium tuberculosis* Utilizing the Recombinase Aided Amplification-Based CRISPR-Cas13a System

**DOI:** 10.3390/microorganisms12081507

**Published:** 2024-07-23

**Authors:** Qiao Li, Nenhan Wang, Mengdi Pang, Honghao Miao, Xiaowei Dai, Bo Li, Xinyu Yang, Chuanyou Li, Yi Liu

**Affiliations:** 1 Biobank of Beijing Chest Hospital, Capital Medical University/Beijing Tuberculosis & Thoracic Tumor Research Institute, Beijing 101149, China; 2 Beijing Center for Disease Prevention and Control, Beijing 100013, China; 3 School of Public Health, Capital Medical University, Beijing 100069, China

**Keywords:** *Mycobacterium tuberculosis*, recombinase aided amplification, CRISPR-Cas13a, clinical diagnosis

## Abstract

Tuberculosis (TB), a disease caused by *Mycobacterium tuberculosis* (MTB) infection, remains a major threat to global public health. To facilitate early TB diagnosis, an IS6110 gene-based recombinase aided amplification (RAA) assay was coupled to a clustered, regularly interspaced short palindromic repeats (CRISPR)-Cas13a fluorescence assay to create a rapid MTB detection assay (named RAA-CRISPR-MTB). Its diagnostic efficacy was evaluated for sensitivity and specificity through sequential testing of recombinant plasmids, mycobacterium strains, and clinical specimens. RAA-CRISPR detected IS6110 genes at levels approaching 1 copy/μL with pUC57-6110 as the template and 10 copies/μL with H37Rv as the template. There was no observed cross detection of non-tuberculosis mycobacteria (NTM) with either template. Furthermore, RAA-CRISPR testing of 151 clinical specimens yielded a diagnostic specificity rate of 100% and a diagnostic sensitivity rate of 69% that exceeded the corresponding Xpert MTB/RIF assay rate (60%). In conclusion, we established a novel RAA-CRISPR assay that achieved highly sensitive and specific MTB detection for use as a clinical TB diagnostic tool in resource-poor settings.

## 1. Introduction

Tuberculosis (TB), an infectious disease caused by *Mycobacterium tuberculosis* (MTB), remains a major threat to humanity, with approximately 10.6 million incident cases and 1.3 million deaths reported worldwide in 2022 [1]. In order to reduce global TB incidence and prevalence rates, rapid and accurate diagnostic TB tests are a prerequisite for achieving effective control and treatment of this serious disease. Currently, TB diagnosis is based on clinical symptoms, thoracic imaging findings, and laboratory test results. Due to their low costs and easy implementation, sputum smear microscopy and MTB culture are the predominant diagnostic laboratory tests used for clinical TB diagnosis. However, these assays have limitations, such as low sensitivity, poor specificity, and long turnaround times [2,3]. To address these limitations, the Xpert MTB/RIF assay (Xpert) was developed and is currently the most commonly used World Health Organization (WHO)-recommended diagnostic test. Nonetheless, this assay is underutilized in countries and communities with limited resources due to its technical complexity and high cost per test [4,5,6]. Thus, new methods that can achieve rapid screening and diagnosis of TB patients are urgently needed.

Clustered regularly interspaced short palindromic repeats (CRISPR)-associated proteins (Cas) are adaptive defense systems used by bacteria and archaea to combat invasive plasmids and viruses [7,8]. Key Cas family members include Cas9, which has been successfully used for gene editing, and Cas12 and Cas13, which have been used in vitro to detect various pathogens based on their collateral cleavage activities [9,10,11]. Cas13 possesses two distinct domains: a recognition lobe domain that recognizes and binds to pre-crRNA and a nuclease lobe domain that cleaves target RNA guided by crRNA. Notably, the CRISPR-Cas13 system has been incorporated as part of a highly sensitive assay for detecting Zika and Dengue viruses with a low detection limit of 2 aM [12], which prompted us to develop a comparably sensitive MTB detection assay. In fact, we previously developed an excellent diagnostic assay incorporating a CRISPR-Cas system and a PCR amplification method for use in detecting the MTB IS1081 sequence in clinical specimens [13].

Recombinase aided amplification (RAA) is a novel isothermal nucleic acid amplification technology. It does not require precisely controlled thermal cycling to achieve amplification, which has prompted its widespread use as molecular diagnostic assays for rapid nucleic acid sequence-based detection of several pathogens in non-clinical laboratories [14]. In the current study, we established a highly sensitive and specific MTB detection method incorporating the CRISPR-Cas13a system and RAA methods (hereinafter referred to as RAA-CRISPR-MTB) for use in detecting the MTB IS6110 sequence in place of the IS1081 sequence detected in our previous reported assay in order to improve MTB detection sensitivity and specificity. The RAA-CRISPR method was comprehensively evaluated for MTB detection performance through successive testing of plasmids, MTB and non-MTB strains, and clinical specimens, and then sensitivity and specificity rates were compared to corresponding rates obtained using other diagnostic assays (e.g., smear, MTB culture, and Xpert).

## 2. Materials and Methods

### 2.1. Patients and Samples Collection

Sputum specimens were collected from 360 suspected active TB patients who were diagnosed at the Beijing Center for Tuberculosis Research and Control from 1 June 2021 to 30 June 2022. From each patient with suspected pulmonary TB, three sputum specimens were collected then tested separately via MTB smear testing, MTB culture, and Xpert, with some samples tested via RAA-CRISPR-MTB. Final clinical diagnoses were formulated by attending physicians based on clinical symptoms, thoracic imaging findings, and laboratory test results. All samples were stored at −80 °C until assayed. The design of the study and all study procedures were approved by the Ethics Committee of Beijing Chest Hospital, Capital Medical University (approval number: YJS-2021-027). Exclusion criteria included (1) incomplete clinical data, (2) lack of smear/culture/Xpert results, and (3) submission of unsuitable specimens for RAA-CRISPR-MTB.

### 2.2. DNA Rapid Extraction

Sputum specimens were treated with Sample Reagent (Cepheid Inc., Sunnyvale, CA, USA) (*v*/*v* = 1:1) for 30 min at room temperature. After 15 min of centrifugation at 13,800× *g*, the resulting pellet was resuspended in 50 μL of RNase-free water (TransGen Biotech Co., Ltd., Beijing, China), then the suspension was heated for 20 min at 100 °C. Thereafter, 2 μL of heated lysate was used as the template for the RAA-CRISPR-MTB assay.

### 2.3. Preparation of the MTB Gene and H37Rv DNA

The positive control plasmid template for RAA-CRISPR-MTB validation was prepared by synthesizing a DNA fragment based on a partial MTB IS6110 gene sequence (314 bp) that was inserted into the pUC57 vector (2710 bp) to construct the recombinant plasmid pUC57-6110 (Sangon Co., Ltd., Shanghai, China). The pUC57-6110 plasmid was confirmed to contain the correct DNA insert via enzyme digestion and DNA sequencing. Next, the concentration of recombinant pUC57-6110 was converted from (X) ng/μL to (Y) copies/μL using the formula ((Y) copies/μL = 6.02 × 10^23^ × (X) ng/μL × 10^−9^/(Z) bp × 660), then a diluted plasmid stock solution (10^0^–10^7^ copies/μL) was used as the standard template for MTB detection.

MTB strain DNA templates for RAA-CRISPR-MTB validation were each prepared from a single colony scraped from the surface of a Lowenstein–Jenden solid medium culture (Celnovte Biotech, Zhengzhou, China) that was suspended in a volume of RNase-free water to achieve an optical density (OD_600_) value of 1 (1 OD_600_ = 3.2 × 10^9^ copies/mL). Thereafter, DNA was extracted from the diluted suspension (10^0^–10^6^ copies/μL) using the same method as described above for sputum DNA extraction.

### 2.4. Preparation of Cas13a Protein

CRISPR-Cas13a system components (Cas protein and crRNA) were prepared in-house. Briefly, for Cas protein preparation, Twinstrep-SUMO-huLwCas13a (Addgene, Watertown, MA, USA) expression was induced by the addition of IPTG to Rosetta (DE3) Competent Cells (CoWin Biosciences, Cambridge, MA, USA), and then the LwCas13a protein was purified in successive steps via nickel (Ni^2+^) column chromatography followed by small ubiquitin-like modifier (SUMO) protease digestion and cation exchange column chromatography. All Cas protein expression and purification procedures were conducted as previously described [15].

### 2.5. Preparation of CrRNA

For crRNA preparation, the crRNA sequence was first synthesized as single-stranded DNA then was amplified by PCR to incorporate the T7 promoter. The 50 μL PCR mixture contained 25 μL of 2 × Ex Taq Mix (Takara Biotech, Dalian, China), 0.4 µM of forward and 0.4 µM of reverse primer, and 2 μL of DNA template. PCR cycling conditions were as follows: initial denaturation for 3 min at 98 °C, followed by 35 cycles of (98 °C for 10 s, 56 °C for 30 s and 72 °C for 60 s), and a final elongation step of 72 °C for 5 min. Next, the PCR product was purified using DNA Extraction Reagent (Enol: Chloroform = 25:24, pH > 7.8) (Solarbio, Beijing, China); then it was transcribed into single-stranded RNA using T7 RNA polymerase mix.

### 2.6. RAA Coupled with CRISPR-Cas13a Assay

Sample DNA templates were amplified via RAA, and then the detection of the MTB IS6110 DNA sequence was conducted using a CRISPR-Cas13a system-based fluorescent assay. Briefly, each RAA reaction was prepared in a final volume of 50 μL using a commercial kit, Basic Nucleic Acid Amplification Reagent [15] (RAA Method) (Hangzhou ZC Bio-Sci & Tech Co., Ltd., Hangzhou, China). The 50 μL reaction contained 2 μL of sample, 0.4 µM of forward and 0.4 µM reverse primer, 1 × reaction buffer, and 1 × freeze-dried reaction pellets. The reaction was performed with 14 mM magnesium acetate in a final volume of 50 μL according to the manufacturer’s instructions, then incubated for 30 min at 39 °C.

Thereafter, the RAA product (or RNase-free water as a negative control) was added to the CRISPR-Cas reaction mix containing the final concentrations of components: 5 µL RAA products, 1 IU/µL T7 RNA polymerase Mix, 1.6 IU/µL RNase inhibitor, and 2.5 mM ribonucleoside triphosphates (New England Biolabs, Ipswich, MA, USA), 2 nM reporter RNA (Thermo Fisher Scientific, Waltham, MA, USA), 2 µM crRNA, 25 nM Cas13a, and 10 × Nuclease assay buffer (400 mM Tris-HCl, 600 mM NaCl, and 60 mM MgCl_2_). The reactions were performed at 37 °C using the 7500 Real Time PCR System (Applied Biosystems, Foster City, CA, USA), and then fluorescent signals emitted by products were monitored for 60 min.

### 2.7. Statistical Analysis

The MTB DNA and H37Rv strain results were expressed as copies/μL (template concentration). Statistical analysis and figures were conducted using GraphPad Prism, version 5.0 (GraphPad Software, San Diego, CA, USA). The data were expressed as means ± standard deviation (SD) for each sample in triplicate, using one-way analysis of variance (ANOVA). A *p* value of less than 0.05 was considered statistically significant (* *p* < 0.05, ** *p* < 0.01, and *** *p* < 0.001). 

## 3. Results

### 3.1. Schematic of RAA-CRISPR-MTB

**Figure 1:**
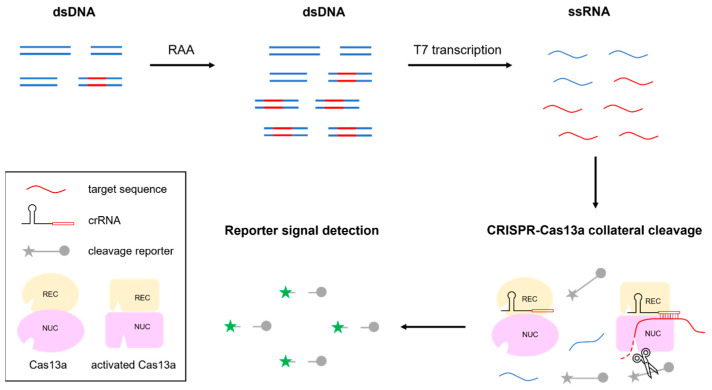
Work principle of CRISPR-based assay for MTB detection. This assay consisted of a target amplification step and detection step. The pretreated samples were conducted to increase the amount of the IS6110 DNA sequence and transcribed into ssRNA, which recognized by specific crRNA, and then generated fluorescent signal.

In this study, we developed a rapid, highly sensitive, and specific MTB detection assay. As depicted in Figure 1, the assay mainly consisted of a target amplification step and detection step. First, pretreated samples served as templates for RAA, which was conducted to increase the amount of the target sequence (MTB IS6110) and to incorporate the T7 promoter at 5′ ends of RAA products. For the subsequent detection step, RAA products were transcribed into single-stranded RNA (ssRNA) using T7 RNA polymerase. Thereafter, the target RNA sequence was recognized and bound by the IS6110 sequence-specific crRNA to trigger collateral Cas13a cleavage in the reporter RNA molecule, which contained a fluorophore on one end and a quencher on the other end. An interaction between fluorophore and quencher at opposite ends of the intact crRNA prevented the fluorophore from generating a fluorescent signal, while cleavage of the reporter molecule triggered by specific crRNA binding to the target sequence resulted in the release of the fluorophore from the quencher. Thereafter, the fluorophore freely entered the reaction solution in an actively fluorescing form, which emitted a bright green signal (upon excitation with light of the appropriate wavelength) that was collected by ABI7500 fluorescent detector.

**Figure 2:**
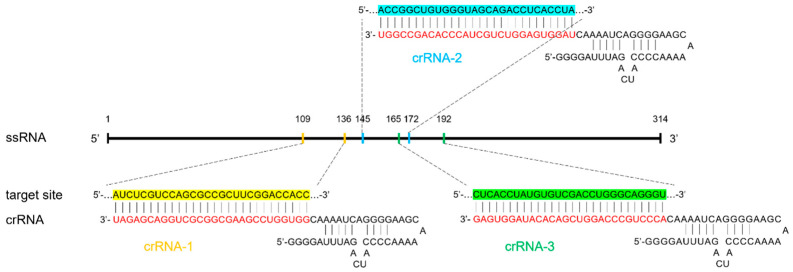
crRNA design for targeting the conserved sequence of IS6110 gene in MTB. CrRNA-1 (yellow), crRNA-2 (blue), and crRNA-3 (green), which target different regions within IS6110 were designed, and their sequences were all divided into two parts, with the guide sequence (28nt, red) recognizing target site and the scaffold sequence (39nt, black) binding to Cas13a.

A strong signal indicated the presence of the IS6110 DNA sequence in the sample, which was selected as the MTB detection target due to its sequence conservation across MTB strains. Meanwhile, three crRNAs targeting different regions within IS6110 were designed (Figure 2, Supplementary Table S1) and screened in our previous study [16]; the one (named crRNA-1) that showed the highest sensitivity was then used throughout this study.

### 3.2. Detection of RAA-CRISPR Method Using the MTB Gene

**Figure 3:**
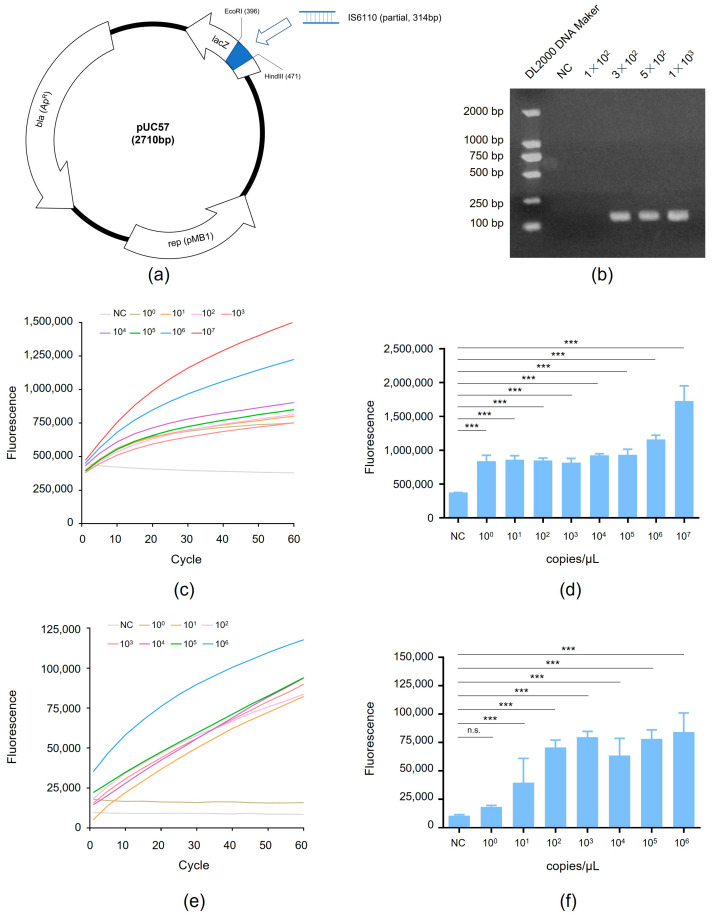
Detection of RAA-CRISPR method using the MTB gene: (**a**) The map of pUC57 plasmid with MTB gene. (**b**) The target sequence in MTB plasmid were amplified by RAA and detected by agarose gel electrophoresis. (**c**) Serial dilutions of the MTB gene were detected by the RAA-CRISPR method. (**d**) The fluorescent intensity of the MTB gene at 60 cycle; (**e**) Serial dilutions of the standard strain H37Rv were detected by the RAA-CRISPR method. (**f**) The fluorescent intensity of the standard strain H37Rv at 60 cycle. Note: *** stands for *p* < 0.001, n.s.: no significance. Error bars represent the means ± SEM from three independent experiments with triplicate samples.

Next, we explored RAA-CRISPR assay sensitivity by testing serial dilutions of a plasmid containing the MTB IS6110 gene that was successfully constructed by our group by inserting a partial IS6110 sequence into the pUC57 vector (Figure 3a, Supplementary Table S2). Using serial dilutions of this plasmid as templates with known IS6110 copy numbers, we tested the RAA-CRISPR assay using samples with 3 × 10^2^ copies/μL, 5 × 10^2^ copies/μL, and 1 × 10^3^ copies/μL (as confirmed via agarose gel electrophoresis), and 1 × 10^2^ copies/μL (which could not be confirmed via agarose gel electrophoresis) (Figure 3b). In addition, RAA-CRISPR testing of serial dilutions of the plasmid revealed that the assay provided high-level sensitivity, due to its low limit of detection of 1 IS6110 copy/μL (Figure 3c,d). Taken together, these results suggest that adding an additional RAA target-amplification step before the CRISPR-Cas13a-based target sequence detection step further increased CRISPR-Cas13a sensitivity that enabled the detection of very low target MTB IS6110 gene copy numbers.

### 3.3. Detection of RAA-CRISPR Method Using the Standard Strain H37Rv

To further verify the abovementioned enhanced RAA-CRISPR detection sensitivity, we assayed serial dilutions of the MTB strain H37Rv positive control and found that the RAA-CRISPR method could detect 10 copies/μL (Figure 3e,f) of the MTB IS6110 target sequence. Therefore, the results indicated that the Cas13a-based assay has advantages in detecting MTB due to its high sensitivity.

### 3.4. Detection of the Specificity of RAA-CRISPR Method

**Figure 4:**
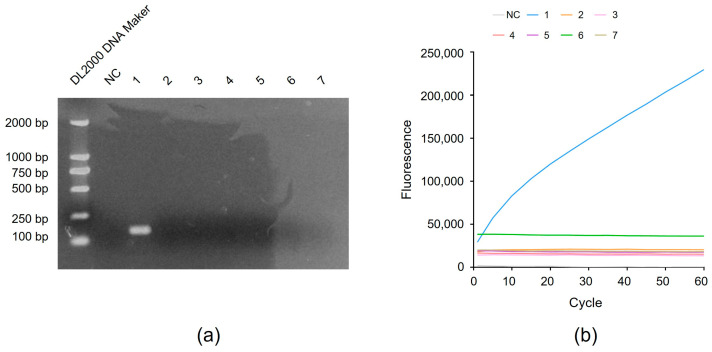
Detection of the specificity of RAA-CRISPR method. The standard strain H37Rv and six kinds of non-tuberculosis mycobacteria were detected by agarose gel electrophoresis (**a**) and RAA-CRISPR method (**b**). 1: H37Rv; 2: *M. kansasii*; 3: *M. gordonae*; 4: *M. avium*; 5: *M. intracellulare*; 6: *M. abscessus*; 7: *M. fortuitum*.

With regard to assay specificity, no samples collected from patients with non-tuberculous mycobacteria (NTM) infections produced false-positive results, as based on comparisons between target DNA band intensities (Figure 4a) and fluorescent signals (Figure 4b) obtained for non-MTB strains versus those obtained for MTB strain H37Rv. Taken together, the abovementioned results indicate that RAA-CRISPR is a highly sensitive and specific MTB-detection method with great clinical potential as a TB diagnostic assay.

### 3.5. Flowchart of the Study Population

**Figure 5:**
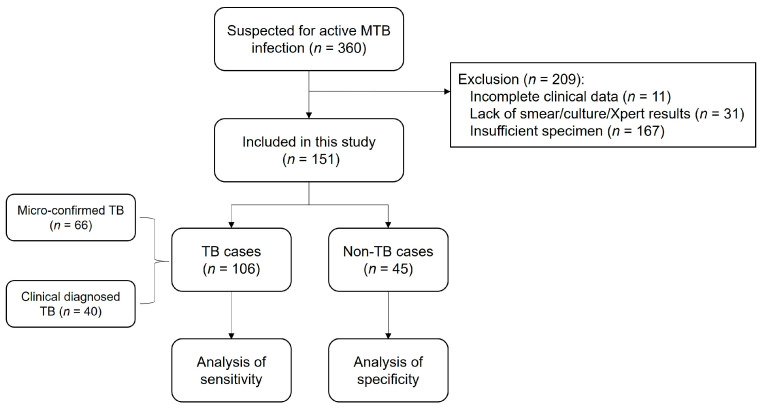
Flowchart of the study population.

From 1 June 2021 to 30 June 2022, 360 patients with suspected active MTB infections were reviewed, of whom 209 were excluded because they lacked the required microbiological test results (smear/culture/Xpert) or were unable to provide suitable specimens for RAA-CRISPR testing. Next, the RAA-CRISPR method was evaluated using specimens obtained from the remaining 151 patients, all of whom provided complete clinical data. This group of patients included microbiologically confirmed TB cases and clinically diagnosed TB and non-TB cases (Figure 5).

### 3.6. Application of RAA-CRISPR in Clinical Tuberculosis

**Figure 6:**
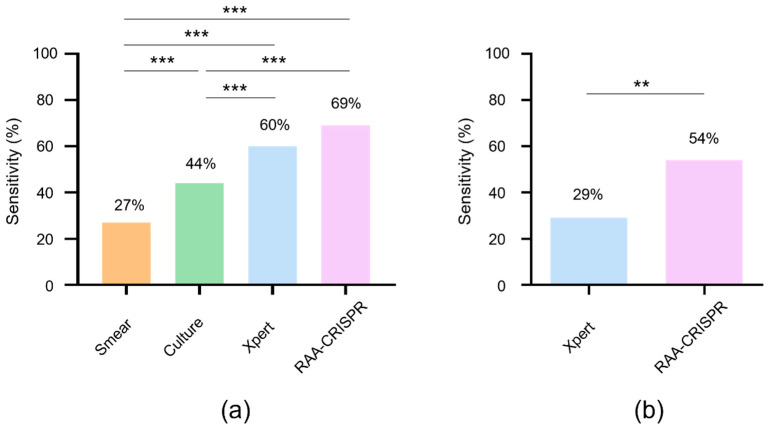
Comparison of RAA-CRISPR, smear, culture, and Xpert methods using clinical samples: (**a**) The diagnostic sensitivity of these methods in 106 TB cases. (**b**) The diagnostic sensitivity of RAA-CRISPR and Xpert in 56 smear- and culture-negative TB cases. McNemar’s test, ** *p* < 0.01, *** *p* < 0.001.

Thereafter, RAA-CRISPR assay results and microbiological assay-based results obtained for the 151 patients were compared. Ultimately, the results of the laboratory testing revealed 106 TB cases, of which 29 cases were detected by smear (27% sensitivity, 29/106), 47 cases by culture (44% sensitivity, 47/106), 64 cases by Xpert (60% sensitivity, 64/106), and 73 cases by RAA-CRISPR (69% sensitivity, 73/106) (Figure 6a). Meanwhile, RAA-CRISPR testing of the 56 smear-negative and culture-negative cases among the 106 TB cases provided a sensitivity rate for MTB detection approaching 54% (30/56), which was significantly higher than that obtained using the Xpert assay (29%, 16/56) (Figure 6b). With regard to specificity, no false-positive RAA-CRISPR results were obtained for 45 non-TB cases, for a MTB-detection specificity rate of 100% (Supplementary Table S4). Taken together, these results suggest that our RAA-CRISPR assay offers significant advantages over other clinical MTB detection assays.

## 4. Discussion

The CRISPR-Cas13 system, an in vitro diagnostic tool based on RNA detection [17], is currently used in numerous clinical applications. These systems are vastly different from the early CRISPR-Cas system discovered by Jennifer Doudna, which provides two distinct Cas RNases that guide RNA processing and detection with high specificity but low sensitivity [18]. Since that time, Zhang has developed the Cas13a-based molecular detection platform (SHERLOCK), then combined this platform with isothermal amplification techniques to create a method that could detect specific strains of Zika and Dengue virus with attomolar-level sensitivity [12,19,20]. Notably, this technology has been increasingly adopted for the detection of SARS-CoV2 during the COVID-19 pandemic due to its adaptability to delivery as fluorescence readout-based lateral flow test strips to facilitate population screening [21,22]. As such, the CRISPR-Cas13 platform holds promise as an effective detection method for use in diagnosing infectious diseases caused by RNA viruses [23,24], DNA viruses [25,26], and bacteria [27].

In this study, we established the RAA-CRISPR assay, a highly sensitive and specific MTB detection assay based on the CRISPR-Cas13 system. Importantly, the IS6110 insertion sequence is exclusively found in the genomes of MTB and other members of the *M. tuberculosis* complex (MTBC) as multiple copies of IS6110 per genome and thus can enhance MTB detection sensitivity and specificity better than assays based on single-copy gene detection [28,29,30]. Therefore, here, IS6110 was used to construct the recombinant plasmid-based standard template that was used to preliminarily validate the RAA-CRISPR method and demonstrate the single IS6110-copy detection sensitivity of the method. Furthermore, RAA-CRISPR testing of the H37Rv MTB strain provided a sensitivity rate of ten copies/μL and thus was less sensitive than the RAA-CRISPR testing of the recombinant plasmid; a result that was presumably due to the relatively lower purity of the H37Rv IS6110 target sequence. The H37Rv strain is currently the most commonly used model strain in tuberculosis research, but it is not the strain that causes the most severe human infections. Given the high sensitivity of the RAA-CRISPR assay and the conserved nature of the IS6110 sequence, we will continue to study whether this assay can be applied to detect strains that cause severe tuberculosis. Additionally, the RAA-CRISPR method provided significant specificity for distinguishing between MTB and NTM.

In addition to its abovementioned advantages of high sensitivity and specificity, RAA-CRISPR is a simple and rapid MTB detection method that can be completed within 1.5 h. RAA-CRISPR comprises two steps: the RAA step conducted at 39 °C for 30 min and the Cas13a-based detection step conducted at 37 °C for 60 min. Since both steps are performed at almost the same temperature, we attempted to combine both steps into a single step to reduce the risk of contamination, resulting in less-than-ideal results. Therefore, in future experiments we plan to optimize the ratio of reaction components and prepare these components as a freeze-dried powder to improve the suitability of this method for use in poor-resource settings, where appropriate equipment and reagents are unavailable. Moreover, as compared to Cas12a, which cleaves single-stranded DNA [31,32,33] (ssDNA), our RAA-CRISPR method incorporates a transcription step after the isothermal RAA step that increases RAA-CRISPR detection sensitivity by boosting detection signal strength.

For pulmonary TB diagnosis, RAA-CRISPR provides comparable diagnostic efficacy to that obtained via Xpert and culture-based assays without producing false-positive results. Most importantly, RAA-CRISPR sensitivity was significantly superior to that of Xpert in smear- and culture-negative TB cases, which is a key advantage of RAA-CRISPR. Furthermore, sputum specimens tested using RAA-CRISPR were chemically treated and boiled during the pretreatment stage to break cell walls to facilitate rapid DNA extraction, which simplified the procedure to thereby render the test more suitable for point-of care testing (POCT). Notably, the estimated cost of our RAA-CRISPR assay of only USD 8.2 per sample may be further decreased if the assay can be adapted for use in batch testing.

Although our results highlight the promising diagnostic value of CRISPR-based MTB detection assays, such methods require specialized equipment and trained professionals and thus are not suitable for use in low-resource settings. To address this issue, our future research will focus on the development of customized lateral flow strips based on our RAA-CRISPR assay design that may provide easily visualized and interpreted results when used in settings other than clinical laboratories.

In conclusion, RAA-CRISPR-MTB is a highly sensitive and specific method for use in MTB detection. Evaluation of RAA-CRISPR assay performance using clinical specimens demonstrated the novel assay provided greater MTB detection sensitivity than that provided by smear- and culture-based MTB detection methods and comparable sensitivity to that provided by Xpert. As a final note, RAA-CRISPR short turnaround time, low sample input requirement, simplicity, and low cost make it a viable alternative to conventional MTB detection assays for use in clinical TB diagnosis, especially in low-resource settings.

## Data Availability

Data are contained within the article.

## Supplementary Materials

The following supporting information can be downloaded at: https://www.mdpi.com/article/10.3390/microorganisms12081507/s1, Table S1: crRNA sequences used in this study; Table S2: IS6110 sequence used for the MTB gene construction; Table S3: RAA primer sequences used in this study; Table S4: Characteristics of enrolled cases.
