# Supplementary material for: Rapid and Highly Sensitive Detection of Mycobacterium tuberculosis Utilizing the Recombinase Aided Amplification-Based CRISPR-Cas13a System
Source: Microorganisms. 2024 Jul 23;12(8):1507. doi: 10.3390/microorganisms12081507 (PMC11356214; doi:10.3390/microorganisms12081507)
Supplement: Supplementary file 1 [file microorganisms-12-01507-s001.zip › microorganisms-3080560-supplementary.pdf]

Supplementary Materials:

Supplementary Table S1. crRNA sequences used in this study.

Name	Sequence (5′→3′)
crRNA-1	GGGGAUUUAGACUACCCCAAAAACGAAGGGGACUAAAAC GGUGGUCCGAAGCGGCGCUGGACGAGAU
crRNA-2	GGGGAUUUAGACUACCCCAAAAACGAAGGGGACUAAAAC UAGGUGAGGUCUGCUACCCACAGCCGGU
crRNA-3	GGGGAUUUAGACUACCCCAAAAACGAAGGGGACUAAAAC ACCCUGCCCAGGUCGACACAUAGGUGAG

Supplementary Table S2. IS6110 sequence used for the MTB gene construction.

CCAGATGCACCGTCGAACGGCTGATGACCAAACTCGGCCTGTCCGGGACCACC CGCGGCAAAGCCCGCAGGACCACGATCGCTGATCCGGCCACAGCCCGTCCCGC CGATCTCGTCCAGCGCCGCTTCGGACCACCAGCACCTAACCGGCTGTGGGTAGC AGACCTCACCTATGTGTCGACCTGGGCAGGGTTCGCCTACGTGGCCTTTGTCACC GACGCCTACGCTCGCAG GATCCTGGGCTGGCGGGTCGCTTCCACGATGGCCAC CTCCATGGTCCTCGAC GCGATCGAGCAAGCCATCTGGACCCGCCAA

Supplementary Table S3. RAA primer sequences used in this study.

Name	Sequence (5′→3′)
RAA-F	AATTCTAATACGACTCACTATAGGAGATGCACCGTCGAACGG CTGATGACCAAAC
RAA-R	CCAGGTCGACACATAGGTGAGGTCTGCTACCCACA

Supplementary Table S4. Characteristics of enrolled cases.

Case No.	Sputum smear	MTB Culture	Xpert	RAA-CRISPR	TB diagnosis
1	-	+	+	+	Micro-confirmed TB
2	-	-	+	+	Micro-confirmed TB
3	+	+	+	-	Micro-confirmed TB
4	+	+	+	-	Micro-confirmed TB
5	-	+	+	+	Micro-confirmed TB

6	+	+	+	+	Micro-confirmed TB
7	+	+	+	+	Micro-confirmed TB
8	+	+	+	+	Micro-confirmed TB
9	+	+	+	+	Micro-confirmed TB
10	+	+	+	+	Micro-confirmed TB
11	-	-	+	-	Micro-confirmed TB
12	-	+	+	+	Micro-confirmed TB
13	-	+	+	+	Micro-confirmed TB
14	-	-	+	+	Micro-confirmed TB
15	-	+	+	+	Micro-confirmed TB
16	-	-	+	+	Micro-confirmed TB
17	-	-	+	-	Micro-confirmed TB
18	-	+	+	+	Micro-confirmed TB
19	-	+	+	+	Micro-confirmed TB
20	-	-	+	+	Micro-confirmed TB
21	-	+	+	+	Micro-confirmed TB
22	-	+	+	+	Micro-confirmed TB
23	+	+	+	-	Micro-confirmed TB
24	-	+	+	+	Micro-confirmed TB
25	-	-	+	+	Micro-confirmed TB
26	-	+	+	+	Micro-confirmed TB
27	+	+	+	+	Micro-confirmed TB
28	-	+	+	-	Micro-confirmed TB

29	+	+	+	+	Micro-confirmed TB
30	-	+	+	+	Micro-confirmed TB
31	+	+	+	+	Micro-confirmed TB
32	+	+	+	+	Micro-confirmed TB
33	-	+	+	+	Micro-confirmed TB
34	-	-	+	+	Micro-confirmed TB
35	+	+	+	+	Micro-confirmed TB
36	+	+	+	+	Micro-confirmed TB
37	-	-	+	+	Micro-confirmed TB
38	+	-	+	+	Micro-confirmed TB
39	-	+	+	+	Micro-confirmed TB
40	+	+	+	+	Micro-confirmed TB
41	+	+	+	+	Micro-confirmed TB
42	+	+	+	+	Micro-confirmed TB
43	+	+	+	+	Micro-confirmed TB
44	+	+	+	+	Micro-confirmed TB
45	+	+	+	+	Micro-confirmed TB
46	+	+	+	+	Micro-confirmed TB
47	-	+	+	+	Micro-confirmed TB
48	-	-	+	+	Micro-confirmed TB
49	+	+	+	+	Micro-confirmed TB
50	+	+	+	+	Micro-confirmed TB
51	+	-	+	+	Micro-confirmed TB

52	-	-	+	+	Micro-confirmed TB
53	+	+	+	+	Micro-confirmed TB
54	-	+	+	+	Micro-confirmed TB
55	-	+	+	+	Micro-confirmed TB
56	-	+	+	-	Micro-confirmed TB
57	-	+	-	-	Micro-confirmed TB
58	+	-	+	+	Micro-confirmed TB
59	+	+	+	+	Micro-confirmed TB
60	+	+	+	+	Micro-confirmed TB
61	-	-	+	+	Micro-confirmed TB
62	-	+	-	-	Micro-confirmed TB
63	-	-	+	+	Micro-confirmed TB
64	-	-	+	-	Micro-confirmed TB
65	-	-	+	+	Micro-confirmed TB
66	-	-	+	+	Micro-confirmed TB
67	-	-	-	+	Clinical diagnosed TB
68	-	-	-	+	Clinical diagnosed TB
69	-	-	-	+	Clinical diagnosed TB
70	-	-	-	-	Clinical diagnosed TB
71	-	-	-	+	Clinical diagnosed TB
72	-	-	-	+	Clinical diagnosed TB
73	-	-	-	+	Clinical diagnosed TB
74	-	-	-	-	Clinical diagnosed TB

75	-	-	-	-	Clinical diagnosed TB
76	-	-	-	-	Clinical diagnosed TB
77	-	-	-	+	Clinical diagnosed TB
78	-	-	-	-	Clinical diagnosed TB
79	-	-	-	-	Clinical diagnosed TB
80	-	-	-	-	Clinical diagnosed TB
81	-	-	-	-	Clinical diagnosed TB
82	-	-	-	-	Clinical diagnosed TB
83	-	-	-	-	Clinical diagnosed TB
84	-	-	-	-	Clinical diagnosed TB
85	-	-	-	+	Clinical diagnosed TB
86	-	-	-	-	Clinical diagnosed TB
87	-	-	-	-	Clinical diagnosed TB
88	-	-	-	-	Clinical diagnosed TB
89	-	-	-	-	Clinical diagnosed TB
90	-	-	-	-	Clinical diagnosed TB
91	-	-	-	-	Clinical diagnosed TB
92	-	-	-	-	Clinical diagnosed TB
93	-	-	-	+	Clinical diagnosed TB
94	-	-	-	+	Clinical diagnosed TB
95	-	-	-	-	Clinical diagnosed TB
96	-	-	-	-	Clinical diagnosed TB
97	-	-	-	+	Clinical diagnosed TB

98	-	-	-	-	Clinical diagnosed TB
99	-	-	-	-	Clinical diagnosed TB
100	-	-	-	+	Clinical diagnosed TB
101	-	-	-	+	Clinical diagnosed TB
102	-	-	-	-	Clinical diagnosed TB
103	-	-	-	+	Clinical diagnosed TB
104	-	-	-	+	Clinical diagnosed TB
105	-	-	-	+	Clinical diagnosed TB
106	-	-	-	+	Clinical diagnosed TB
107	-	-	-	-	Non-TB
108	-	-	-	-	Non-TB
109	-	-	-	-	Non-TB
110	-	-	-	-	Non-TB
111	-	-	-	-	Non-TB
112	-	-	-	-	Non-TB
113	-	-	-	-	Non-TB
114	-	-	-	-	Non-TB
115	-	-	-	-	Non-TB
116	-	-	-	-	Non-TB
117	-	-	-	-	Non-TB
118	-	-	-	-	Non-TB
119	-	-	-	-	Non-TB
120	-	-	-	-	Non-TB

121	-	-	-	-	Non-TB
122	-	-	-	-	Non-TB
123	-	-	-	-	Non-TB
124	-	-	-	-	Non-TB
125	-	-	-	-	Non-TB
126	-	-	-	-	Non-TB
127	-	-	-	-	Non-TB
128	-	-	-	-	Non-TB
129	-	-	-	-	Non-TB
130	-	-	-	-	Non-TB
131	-	-	-	-	Non-TB
132	-	-	-	-	Non-TB
133	-	-	-	-	Non-TB
134	-	-	-	-	Non-TB
135	-	-	-	-	Non-TB
136	-	-	-	-	Non-TB
137	-	-	-	-	Non-TB
138	-	-	-	-	Non-TB
139	-	-	-	-	Non-TB
140	-	-	-	-	Non-TB
141	-	-	-	-	Non-TB
142	-	-	-	-	Non-TB
143	-	-	-	-	Non-TB

144	-	-	-	-	Non-TB
145	-	-	-	-	Non-TB
146	-	-	-	-	Non-TB
147	-	-	-	-	Non-TB
148	-	-	-	-	Non-TB
149	-	-	-	-	Non-TB
150	-	-	-	-	Non-TB
151	-	-	-	-	Non-TB

-: Negative for MTB detection; +: Positive for MTB detection.
